# Sexual Behaviors and Its Predictors among Iranian Women Living in Kashan City 2017: A Cross-Sectional Study 

**DOI:** 10.22074/ijfs.2018.5228

**Published:** 2018-06-20

**Authors:** Zahra Karimian, Fatemeh Atoof, Seyed Ali Azin, Raziyeh Maasoumi, Effat Merghati Khoei

**Affiliations:** 1 Department of Midwifery, Faculty of Nursing and Midwifery, Kashan University of Medical Sciences, Kashan, Iran; 2 Autoimmune Diseases Research Center, Kashan University of Medical Sciences, Kashan, Iran; 3 Department of Epidemiology and Biostatistics, Kashan University of Medical Sciences, Kashan, Iran; 4 Reproductive Biotechnology Research Center, Avicenna Research Institute (ACECR), Tehran, Iran; 5 Department of Reproductive Health, School of Nursing and Midwifery, Tehran University of Medical Sciences, Tehran, Iran; 6 The Iranian National Center for Addiction Studies (INCAS), Institution for Risk Behavior Reduction, Director of Family and Sexual Health Division, Brian and Spinal Cord Injury Research Center, Neuroscience Institution, Tehran University of Medical Sciences, Tehran, Iran

**Keywords:** Iran, Sexual Behaviors, Women

## Abstract

**Background:**

Women’s sexual well-being has been the center of attention in the field of sexology. Study of sexual behavior and investigating its predictors are important for women’s health promotion. This study aimed to explore the components of women’s sexual behaviors and their possible associations with demographic variables.

**Materials and Methods:**

This study was a cross-sectional study (descriptive and analytic) that was conducted in Kashan city, Iran. A National Sexual Behavior Assessment Questionnaire was completed by 500 women of 15 to 49 who referred to the public health centers. To analyze the data, R software was used, ANOVA or Kruskal-Wallis (for parametric or nonparametric data, respectively) were used to compare outcomes among different groups. In order to evaluate the correlation between the subscales, the Pearson correlation coefficient was used.

**Results:**

From all participants, 31.8% obtained high scores in the sexual capacity, 21.2% had high scores in sexual motivation and 0.2% had high scores in sexual function. In sexual script component, 86.2% of women who held traditional beliefs toward sexual behaviors; the majority (91.5%) of women believed in mutual and relational sexuality, 83.4% believed in androcentricity (male-dominated sexuality). Pearson correlation test showed a significant positive correlation between sexual capacity, motivation, function and sexual script. Linear Regression model showed that sexual capacity is associated with women’s education and age of her spouse. Sexual function and sexual motivation were significantly associated with the age of subjects' spouses.

**Conclusion:**

In this study, subjects had low scores in sexual performance while higher scores were achieved in sexual capacity and motivation. This discrepancy can be attributed to the role of sexual scripts dominating the participants’ sexual interactions in this study. We suggest gender-specific and culturally-sensitive education should become a part of women’s health programs in Iran.

## Introduction

In recent years, researchers have focused on the topic of sexual well-being (1-4). In this regard, sexual behaviors as the main aspect of one’s sexuality have been investigated in order to comprehensively assess one’s sexuality-related needs (5-7). Though there has been extensive research on sexual well-being, attention to women’s sexual well-being is increasing (8-10) because gender-specific approaches have become center of focus in sexuality education programs (11, 12). Gender-specific approaches are seminal; particularly in societies where sexuality is dominantly gendered (13). According to the literature, sexual behaviors do not only refer to erotic behaviors but they involve gender role as well as reproductive and life enhancing behaviors. Hilbert pointed out three main factors namely, sexual capacity (i.e. what person can do), sexual motivation (i.e. what person wants to do); and sexual performance (i.e. what person does), which all affect human’s sexual behaviors (14). Human sexual behavior includes all physical-biological aspects, life experiences, knowledge, attitudes and behaviors, and is influenced by several factors such as physical, physiological, interpersonal and cultural issues (15).

Despite the increasing attention towards sexual well-being of women in particular, it is noteworthy that not only primary sexual health assessment is being ignored, but also basic sex education is completely absent in females’ health programs in Iran (15, 16). This should be considered a major shortcoming since sexual behaviors are highly regarded as essential elements of women’s capacities that influence their marital happiness (17, 18). In some studies presented the discourses and symbolic representations through which the women stated their own experiences about womanhood with focusing on sexuality in the context of marriage (19-21). They explicitly reveled a strong link between women’s sexuality and marital happiness:

"Despite many opportunities available for frank discussion on sexual topics in the context of marriage, penetrating the individuals’ silence was much harder than I expected. Perhaps, I had underestimated the power of the marriage. The crucial point is that open discussion on sexuality and conceptualization of actual sexual activity, were possible only within the context of marriage"(19).

This argument is also confirmed by results of several Iranian empirical research (15, 22). These studies have demonstrated that a positive evaluation of one’s sexual behavior is closely associated with her/his overall sexual well-being. Given the facts that sexuality is considered as an integral part of women’s well-being and their marital happiness, sexual behaviors should be evaluated by a comprehensive sexual well-being assessment, in practice; however, this step has not yet been taken in Iranian clinical settings. This current situation entails the challenge that sexual health care and sexual well-being of women are simply disregarded when developing policies, making decisions and delivering care fora general promotion of subjective well-being and quality of life among women and families.

Despite extensive studies, there are some challenges (i.e. the approach that should be used for assessment of sexual behaviors is not clear) in studies undertaken in the field of women’s sexual behaviors (17).

A study on women’s sexual behavior was conducted by Avasti et al. (23) in India. In this study, vaginal sexual activity was the most common form of sexual activity and oral sex had the lowest prevalence; also, 27% of women reported one problem (such as headaches) or more following having sex. Another study done by Hashemi et al. (24) investigated certain sexual behaviors in women of reproductive age. The results of this study indicated that although oral and anal sex is taboos in Iran, but they have a high prevalence among the couples.

We used this survey to examine women’s sexual behaviors by exploring various aspects of their sexual behaviors and also investigating possible associations between sexual behaviors and demographic variables. The purpose of this study was to explore the components of sexual behaviors and their possible associations with the demographic variables.

## Materials and Methods

This study was a cross-sectional survey (descriptive and analytic) aimed to identify and analyze sexual behaviors among women of reproductive age in Kashan, Iran from June to October 2015. A random sample of 500 women of reproductive age who referred to health centers (14 centers) in Kashan and had the inclusion criteria according to random numbers table was included in this survey. Based on Shirpak et al. (16) study, considering d=1, α: 5 %, σ: 10/9, sample size was calculated 500.

Inclusion criteria included having Iranian nationality and the ability to read and write, and being within the age range of 15-49 years and married as well as having an active sex life. Exclusion criteria comprised of unwillingness to answer questions because of the high number of questions, as well as having medical and surgical conditions.

Research tool in this study was women’s sexual behavior assessment questionnaire developed by Qoreshi et al. (25). Psychometric properties of this questionnaire had been assessed in Iranian women of reproductive age. Theoretical framework of this tool is based on the Hilbert’s classification of sexual behaviors. This questionnaire was designed in four Likert sub-scales that were consisted of thirty-three items. The first sub-scale (ten questions) measures sexual capacity. The second sub-scale that consists of nine questions is the sub-scale of sexual performance. The third sub-scale that has the highest number of items (eleven questions) deals with sexual motivation and the fourth sub-scale (three questions) is sexual script. Sexual script includes guides for sexual thinking, feeling and behavior in different situations (14); here, three scripts were considered based on these questions: the religious-sexual script, the right relational script and the male-dominated script.

Each question has a value of zero to five; So, the minimum and maximum scores for sexual capacity are 0 and 50, for sexual motivation are 0 and 45, for sexual function are 0 and 55 and for sexual script are 0 and 15, respectively. Each of these sub-groups was divided into three parts (<33%, 34-66%, and>67%) based on the obtained scores.

After obtaining written informed consents, the questionnaire was completed by the participants. To analyze the data, R software was used for descriptive statistical analysis to describe the participants and The Kolmogorov-Smirnov test was performed to test the normality of continues variables. Data on capacity, motivation and performance variable were found normally distributed, but not the script variable. Moreover, ANOVA was used to compare outcomes among different groups in terms of capacity, motivation and performance variables and Kruskal-Wallis was used for comparing the means of script variable.

In order to evaluate the correlation between the subscales, the Pearson correlation coefficient was used. In addition, we applied the linear regression model to examine the effect of variables on sexual capacity, function and motivation. Also, Bonferroni method was used the post-hoc test with alpha error correction. For checking whether the data fit the model, we examined the normality of residuals. In addition, R^2^ values were mentioned for each of the models (R^2^ was 0.66 for capacity, 0.78 for motivation and 0.58 for performance). For modeling, first we evaluated the relationship of each risk factor (the age of spouse, level of education and other demographic variable) with the outcome variables, variables with a P<0.3 were used in the regression model. The final model was obtained using backward method considering the P<0.05.

This study was approved by Ethic Committee of Tehran and Shahroud University of Medical Sciences in 2015 (IR.SHMU.REC.2015.44 and TUMS 34262-159-01-96). It should be mentioned that we received informed consent from all participants.

## Results

More than half of the women participated in this study (51.9%) were in the age range of 26 to 35 years old. The majority of women (82.8%) were homemaker; also, the majority of them (39.9%) had a high-school education. About half of the women (54.6%) had a middle economic level. The mean duration of marriage was 10.2 ± 7.7 (Table 1).

**Table 1:** Demographic characteristics of individuals participated in this study


Variable		n (%)

Education		
	Elementary	129 (25.7)
	High school	200 (40)
	University	172 (34.4)
Employment status		
	Homemaker	414 (82.8)
	Employed	86 (17.2)
Level of education of the spouse		
	Elementary	147 (29.3)
	High school	179 (35.7)
	University	175 (35.0)
Employment status of the spouse		
	Self-employed	266 (53.2)
	Worker	104 (20.9)
	Employee	117 (23.3)
	Retired	13 (2.7)
Socioeconomic status		
Very bad		22 (4.4)
	Bad	57 (11.5)
	Intermediate	273 (54.6)
	Good	113 (22.7)
	Very good	35 (7.1)
Age (Y), mean ± SD (30.5 ± 7.3)		
15-25		148 (29.6)
	26-35	260 (51.9)
	36-45	92 (18.5)
		
Age of spouse (Y), mean ± SD (34.8 ± 7.9)		
	20-25	48 (9.6)
	26-35	254 (50.7)
	36-45	150 (30)
	46-55	42 (8.4)
	>56	7 (1.3)
		
Duration of marriage (Y), mean ± SD (10.2 ± 7.7)		
	≤- 5	174 (34.8)
	6-10	128 (25.5)
	11-15	91 (18.2)
	>15	108 (21.6)


The scores of sexual behavior in terms of different subgroups. Most women obtained a medium score in terms of sexual capacity and sexual motivation but a low score for sexual function (<18.3 out of 55) (Table 2).

**Table 2:** The scores of sexual behavior according to the subgroups


Subgroups of sexual behavior (minimum score-maximum score)		n (%)	Mean ± SD

Sexual capacity (0-50)			
	<16.6	39 (7.8)	29.4 ± 8.1
	16.7-33.2	302 (60.4)	
	33.3- 50	159 (31.8)	
Sexual performance (0-55)			
	<18.3	317 (63.4)	16.5 ± 6.6
	18.4-36.6	182 (36.3)	
	36.7-55	1 (0.2)	
Sexual motivation (0-45)			
	<15	28 (5.5)	25.7 ± 6.6
	16-30	366 (73.3)	
	31-45	106 (21.2)	
Sexual script (0-15)			
	<5	28 (5.5)	12.2 ± 3.4
	6-10	366 (73.3)	
	11-15	106 (21.2)	
Sexual behavior (0-165)			
	<55	29 (5.7)	85 ± 1.6
	56-110	442 (88.6)	
	111-165	29 (5.7)	


Pearson correlation test showed a significantly positive correlation between the scores of sexual capacity, motivation and sexual (P<0.001). This means with an increase in one score, the scores of other domains also increase.

The majority of participants agreed with three questions of sexual script (Table 3). Question 1 measured the sexual-religious pattern and Question 2 was a correct belief regarding sexual relationship but question 3 was a wrong belief about the sexual relations between a husband and a wife. Pearson correlation test showed a significant relationship between each of the questions of script and sexual function (P<0.001), sexual motivation and sexual capacity; in this regard, there was a positive and significant relationship between sexual behavior and questions 1 and 2 and a significantly negative relationship between sexual behavior and question 3.

The results of the regression model that assessed the association between sexual capacity, motivation and performance and independent variables (Table 4). As shown in this Table, education and the age of spouse had a significant effect on mean score of sexual capacity. Sexual function had a positive and significant relationship only with the age of spouse and women’s sexual function declined with increasing age of their husbands. Age of woman and age of spouse had a significant correlation with sexual motivation so that with increasing age of women, sexual motivation increased while it declined with increasing age of the husband.

**Table 3:** Distribution of answers to questions of sexual script


Numbers of questions	Items	Mean ± SD	I don’t know n (%)	None n (%)	Low n (%)	Medium n (%)	High n (%)	Very high n (%)

31	Sexual-religious idea	3.9 ± 1.4	28 (5.9)	19 (4)	19 (4)	57 (12)	106 (22.3)	247 (51.9)
32	A right idea regarding sexual relationship	4.3 ± 1.1	7 (1.4)	11 (2.3)	24 (4.9)	35 (7.2)	115 (23.6)	296 (60.7)
33	Traditional andmale-centered idea	3.9 ± 1.5	24 (4.9)	44 (9)	13 (2.7)	46 (9.4)	97 (19.9)	264 (54.1)


*; P<0.05 was considered significant statistically.

**Table 4:** The results of the regression model (outcome: sexual capacity, performance, motivation)


		Standardized coefficients		Unstandardized coefficients	P value
		Std. error	Beta	Beta	

Sexual capacity					
	Education (constant)	1.528	-	29.416	<0.001
	Elementary (base line)	-	-	-	-
	High school	1.094	0.172	2.854	0.000
	University	1.152	0.259	4.392	<0.001
Age of spouse (Y)					
	20-25 (base line)	-	-	-	-
	26- 35	1.411	-0.127	-2.064	0.14
	36-45	1.507	-0.239	-4.300	0.000
	>45	1.907	-0.144	-4.021	0.03
Sexual performance					
	Age of spouse (Y) (constant)	1.043	-	18.405	<0.001
	20-25 (base line)	-	-	-	-
	26- 35	1.146	-0.138	-1.763	0.12
	36-45	1.206	-0.184	-2.560	0.034
	>45	1.465	-0.196	-4.090	0.000
Sexual motivation					
	Age (Y) (constant)	1.098	-	26.598	<0.001
	20-25 (base line)	-	-	-	-
	26- 35	1.325	-0.044	-0.565	0.67
	36-45	1.572	-0.236	-3.383	0.030
	>45	2.438	-0.322	-7.610	0.000
Age of spouse (Y)					
	15-25 (base line)	-	-	-	-
	26-35	0.970	0.094	1.224	0.20
	36 -45	2.930	0.194	7.012	0.01
	>45	1.531	0.138	2.450	0.11


*; P<0.05 was considered significant statistically.

## Discussion

The main objective of this study was to explore the components of sexual behaviors and their possible associations with demographic variables. Our results showed that the majority of women participating in this study obtained a medium score for sexual capacity and sexual motivation but a low score for sexual function. Different aspects of sexual behavior showed a significant correlation with some demographic characteristics of women.

Our results are consistent with the results reported by Qoreshi et al. (25) as they found women’s passive sexual function and argued that women’s not using their sexual capacity is compatible with the male-centered theory in which women are considered as passive parts versus men as active parts.

Since the most important factor of an individual’s sexual function is his/her sexual capacity (14), it was found that women do not optimally use their sexual capacity. This can be explained by the role of sexual script dominating the participants’ sexual interactions.

In this study, most participants’ sexual behaviors were originated from right and wrong sexual scripts. The first question (i.e. “giving positive response to the husband’s sexual demands will leads to rewards”) is a religious -sexual question suggesting that women’s sexual script can be affected by religious beliefs. Merghati Khoi et al. (19) also showed that religion is a main factor in women’s sexual self-understanding, and sexual obedience is known as a factor in women’s chastity and self-esteem.

This emphasizes the importance of religion in sex education. Women’s sexual behaviors seem to change through women’s empowerment and educational interventions based on religious norms. Addis et al. (26) showed that patients complain about lack of sex education compatible with culture and religious. In our study, it was identified that the sexual-religious script has a relationship with sexual function, sexual capacity and sexual motivation. Shirpak et al. (16) also found the effect of religion on people’s sexual behaviors.

The second question of sexual script (i.e. "giving a positive response to the husband’s sexual demands creates more intimacy between couples and peace in the family.") is also a right idea regarding sexual relationship that reinforces the relationship between couples. Merghati khoie et al. (19) believed that this emotional connection is an important factor in women’s sexual satisfaction and that should be considered by health care providers. In our study, this script was related to sexual function, sexual motivation and sexual capacity that has been also demonstrated by other studies (25).

But, the third question of sexual script (i.e. "giving a positive response to the husband’s sexual demands prevents the husband’s extramarital relationships.") represents a traditional cultural scenario created by a restricted and male-dominated sexual script. In the qualitative study of Shirpak et al. (15), it was indicated that although many Iranian women have low desire for sex, they do not refuse their husband’s sexual requests. Hashemi et al. (24) showed that one of the reasons that women could not refuse their husbands’ sexual demands was the issue of obedience and women’s obligation to comply with their husbands’ demands, and the other reason was women’s fear of their husbands’ extramarital affairs. In our study, the limited and male-dominated sexual script had a negative relationship with women’s sexual behaviors, which was supported by the study of Qoreshi et al. (25).

In this study, there was a significant relationship between sexual script and women’s sexual behaviors. The results of this study indicated the role of social and cultural determinants in shaping sexual behaviors, which is consistent with the social construction theory (14).

In this study, sexual capacity had a significant association with education level and the age of spouse but it was not associated with woman’s job, economic status and the number of children. In Addis’s study, sexual satisfaction was correlated with age, so that young women were more sexually active and had more sexual satisfaction. In this study, the researcher found that sexual satisfaction reduced with increasing ages (26). Rahmani et al. (1) believed that the cause of decreased sexual satisfaction with increasing age is the interference of sexual activity with other tasks, such as taking care of children, job, etc. Our study identified that education is related to sexual behavior. They also concluded that sexual satisfaction has a relationship with the level of education and the higher the level of education of husbands, the higher women’s sexual satisfaction. In the study of Alavi et al. (27) also, with increasing levels of education, sexual function was improved; authors believed that this occurred as educated people participated in educational programs. In general, in our study, sexual behaviors varied according to women’s demographic characteristics. Billy e al. (28) also showed that people's sexual behaviors vary according to demographic and social characteristics.

Herbenick et al. (29) in their study on sexual behavior of women living in the United States, showed that sexual behaviors markedly varied among women and masturbation was reported to have high prevalence in women aging 18-39 years. In that study, women reported sex with same-sex partners and oral and anal sex in their recent sexual activities. Sexual behaviors in our study was different from other studies that shows that sexual behavior and sexual orientation vary from one society to another due to differences in socio-cultural structure of each society that is consistent with the theory of social construction. According to this paradigm, all human experiences are sociocultural products. This is also true about the issues related to human sexuality; Masters et al. (14) explained the factors affecting sex and noted that in addition to the biology, social and cultural factors have pronounced influences on sex.

Laws and Scwartz (30) also applied social constructionism to explain sexual behaviors in women. They believed that exclusive experiences such as menarche, pregnancy, childbirth, sex, inability to have sex, and sexual dysfunction, in addition to biological basis, were affected by other events.

Finally, premarital sex education is suggested to be implemented for young women to enable them to identify their sexual capacity and sexual motivation, correct the deterrent attitudes and strengthen the reinforcing attitudes for having safe sex. Suitable sexual behaviors can increase marital satisfaction, maintain the foundation of family and ultimately promote the public health.

## Conclusion

This study results showed that the participants’ sexual function is not in at a good level; however, sexual capacity and sexual motivation are reported to be at an acceptable level. This might be due to the role of sexual scripts dominating the participants’ sexual interactions in this study. Premarital education can familiarize women with different dimensions of sexual motivation, sexual capacity and sexual function by taking into account cultural-sexual scenarios. Finally, we suggest assessment of sexual behaviors in other age groups such as adolescents and designing an educational program for promotion of sexual behaviors.
